# Successful treatment of coexisting alopecia areata and hidradenitis suppurativa with upadacitinib^☆^

**DOI:** 10.1016/j.abd.2026.501453

**Published:** 2026-08-31

**Authors:** Patrícia Moreira Gomes, Ruben Costa, Ana Pedrosa, Carmen Lisboa

**Affiliations:** a Department of Dermatology and Venereology, Unidade Local de Saúde São João, Porto, Portugal; b Faculty of Medicine, University of Porto, Porto, Portugal

Dear Editor,

Alopecia areata (AA) and Hidradenitis Suppurativa (HS) are chronic, immune-mediated inflammatory dermatoses.^1^, ^2^ AA is characterized by non-scarring hair loss resulting from immune-mediated targeting of the hair follicle,^1^ and HS is a chronic, relapsing follicular disorder marked by painful nodules, abscesses, and sinus tract formation.^2^ Despite their distinct clinical manifestations, both conditions are driven by dysregulated innate and adaptive immune responses, with proinflammatory cytokines contributing to sustained tissue inflammation.^1^, ^2^ Given the central role of Janus Kinase (JAK)-Signal Transducer and Activator of Transcription (STAT) signaling in multiple cytokine pathways, selective JAK inhibition represents a promising therapeutic strategy.^3^ However, evidence supporting its use, particularly for their concurrent treatment, remains limited.

We report the case of a 24-year-old woman with Down syndrome, hypothyroidism, and overweight who was referred to our dermatology clinic for management of long-standing AA and HS.

HS had its onset during adolescence, followed by a chronic, relapsing course, characterized by recurrent painful inflammatory nodules and abscesses with intermittent drainage, affecting the inguinal region. Over the years, she received multiple courses of systemic antibiotics, including tetracyclines and combined clindamycin plus rifampicin, achieving only partial and temporary improvement. She also underwent surgical excision of HS lesions in the right inguinal region. She was receiving metformin for metabolic management, with no significant impact on HS activity. Regarding AA, the patient first developed localized scalp hair loss at age 16, with complete hair regrowth after treatment with topical and intralesional corticosteroids, topical minoxidil, and topical immunotherapy with diphenylcyclopropenone. At age 23, she experienced recurrence with rapid progression to alopecia universalis.

Physical examination revealed complete loss of scalp hair, eyebrows (Fig. 1A‒C), and body hair. Trichoscopy demonstrated yellow dots and scattered vellus regrowth, without evidence of scarring, consistent with AA. Examination of the inguinal regions revealed multiple inflammatory nodules with early sinus tract formation, consistent with Hurley stage II HS.

**Fig. 1:**
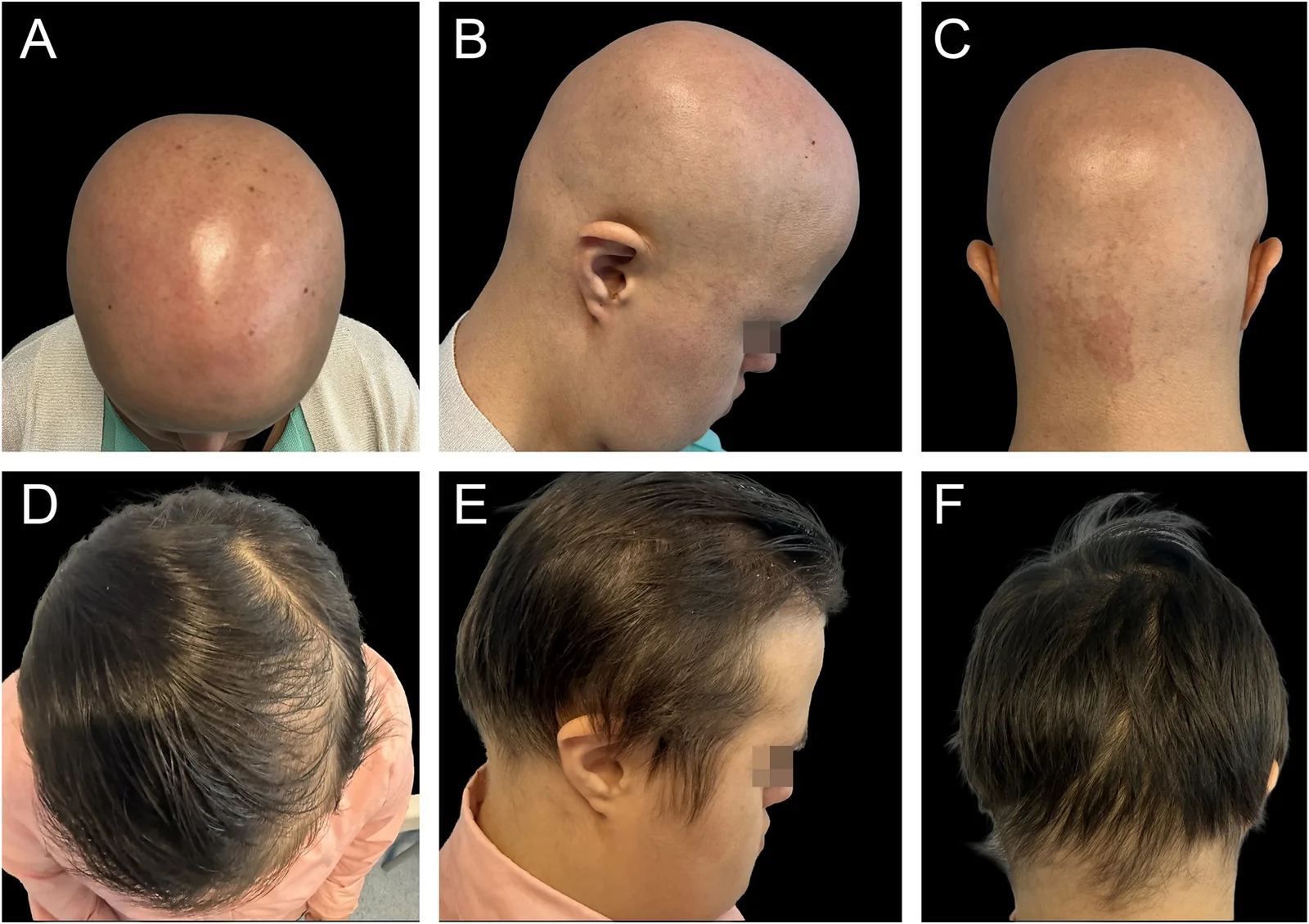
Alopecia areata universalis (SALT 100): (A) Superior; (B) Lateral; (C) Posterior. Near-complete regrowth of scalp and eyebrows (SALT 5) after 9-months of upadacitinib treatment: (D) Superior; (E) Lateral; (F) Posterior.

Given the refractory nature of both diseases and their substantial psychosocial burden, systemic immunomodulatory therapy was considered. After discussing potential risks and benefits, treatment with upadacitinib 15 mg once daily was initiated, with regular clinical and laboratory monitoring. After four months of therapy, marked clinical improvement was observed. The patient achieved Hidradenitis Suppurativa Clinical Response (HiSCR-90), with a marked reduction in inflammatory nodules and no new abscesses or draining fistulas. Concomitantly, partial scalp hair regrowth was noted, with a Severity of Alopecia Tool (SALT) score of 37. At nine months, further improvement was documented, with near-complete scalp regrowth (SALT-5, Fig. 1D‒F) and sustained HS response. The patient and her family reported significant improvement in functional capacity, social interactions, and overall quality of life. No clinically relevant adverse events or laboratory abnormalities were observed during follow-up.

Both AA and HS are immune-mediated disorders characterized by complex inflammatory networks, in which the JAK-STAT pathway represents a key downstream signaling axis.^1^, ^2^ In AA, the collapse of hair-follicle immune privilege leads to cytotoxic T-cell activation, sustained by an Interferon (IFN)-γ/Interleukin (IL)-15 inflammatory loop dependent on JAK signaling.^1^ In HS, inflammation is associated, in part, with upregulation of JAK-dependent cytokines, including IL-1β, IL-17, IL-23, and IL-10, supporting a role for this pathway in disease persistence.^2^, ^4^ By selectively inhibiting JAK1, upadacitinib may modulate multiple inflammatory axes implicated in both conditions.^3^ Clinical evidence supports this rationale. Several JAK inhibitors have demonstrated efficacy in severe AA, with increasing real-world data supporting upadacitinib use.^5^, ^6^ In HS, phase 2 studies have shown promising reductions in inflammatory lesions with JAK1 inhibition, and phase 3 trials are ongoing to further define its therapeutic role.^7^ Evidence regarding dual efficacy in concomitant HS and AA remains limited. Although successful treatment of AA concomitant with other immune-mediated diseases, such as atopic dermatitis,^8^ psoriasis,^9^ and Crohn’s disease,^10^ has been reported with JAK inhibitors, data specifically addressing the coexistence of AA and HS are lacking. To our knowledge, this is the first reported case of concomitant AA and HS successfully treated with upadacitinib. This observation underscores the shared immunologic substrate of both diseases and supports selective JAK1 inhibition as a potential therapeutic strategy in overlapping immune-mediated dermatoses.

## ORCID ID

Patrícia Moreira Gomes: 0000-0003-3981-8961

Ruben Costa: 0000-0003-2376-6837

Ana Pedrosa: 0000-0001-5969-0235

Carmen Lisboa: 0000-0001-8247-8902

## Financial support

None declared.

## Authors’ contributions

Patrícia Moreira Gomes: The study concept and design; data collection, or analysis and interpretation of data; writing of the manuscript or critical review of important intellectual content; critical review of the literature; final approval of the final version of the manuscript.

Ruben Costa: The study concept and design; critical review of the literature and final approval of the final version of the manuscript.

Ana Pedrosa: The study concept and design; effective participation in the research guidance, intellectual participation in the propaedeutic and/or therapeutic conduct of the cases studied, and final approval of the final version of the manuscript.

Carmen Lisboa: The study concept and design; effective participation in the research guidance, intellectual participation in the propaedeutic and/or therapeutic conduct of the studied cases and final approval of the manuscript.

## Research data availability

Does not apply.

## Conflicts of interest

None declared.
